# Spectroscopic Characterization of a Green Copper Site in a Single-Domain Cupredoxin

**DOI:** 10.1371/journal.pone.0098941

**Published:** 2014-06-16

**Authors:** Magali Roger, Frédéric Biaso, Cindy J. Castelle, Marielle Bauzan, Florence Chaspoul, Elisabeth Lojou, Giuliano Sciara, Stefano Caffarri, Marie-Thérèse Giudici-Orticoni, Marianne Ilbert

**Affiliations:** 1 Unité de Bioénergétique et Ingénierie des Protéines, Institut de Microbiologie de la Méditerranée, CNRS-UMR7281, Aix-Marseille Université, Marseille, France; 2 Department of Earth and Planetary Science, University of California, Berkeley, California, United States of America; 3 Unité de Fermentation, Institut de Microbiologie de la Méditerranée, CNRS-FR 3479, Aix Marseille Université, Marseille, France; 4 Unité Chimie Physique, Prévention des Risques et Nuisances Technologiques, Faculté de Pharmacie, CNRS-UMR 7263, Aix-Marseille Université, Marseille, France; 5 Unité de Biologie Végétale et Microbiologie Environnementales, CNRS-UMR 7265, CEA, Aix Marseille Université, Marseille, France; University of South Florida College of Medicine, United States of America

## Abstract

Cupredoxins are widespread copper-binding proteins, mainly involved in electron transfer pathways. They display a typical rigid greek key motif consisting of an eight stranded β-sandwich. A fascinating feature of cupredoxins is the natural diversity of their copper center geometry. These geometry variations give rise to drastic changes in their color, such as blue, green, red or purple. Based on several spectroscopic and structural analyses, a connection between the geometry of their copper-binding site and their color has been proposed. However, little is known about the relationship between such diversity of copper center geometry in cupredoxins and possible implications for function. This has been difficult to assess, as only a few naturally occurring green and red copper sites have been described so far. We report herein the spectrocopic characterization of a novel kind of single domain cupredoxin of green color, involved in a respiratory pathway of the acidophilic organism *Acidithiobacillus ferrooxidans*. Biochemical and spectroscopic characterization coupled to bioinformatics analysis reveal the existence of some unusual features for this novel member of the green cupredoxin sub-family. This protein has the highest redox potential reported to date for a green-type cupredoxin. It has a constrained green copper site insensitive to pH or temperature variations. It is a green-type cupredoxin found for the first time in a respiratory pathway. These unique properties might be explained by a region of unknown function never found in other cupredoxins, and by an unusual length of the loop between the second and the fourth copper ligands. These discoveries will impact our knowledge on non-engineered green copper sites, whose involvement in respiratory chains seems more widespread than initially thought.

## Introduction

Copper is an essential transition element in living organisms. Since the release of oxygen into the atmosphere, copper became available for biological systems, allowing the apparition of a wide range of ubiquitous copper-binding proteins of various functions [1], [2]. Copper proteins have been shown to be key players in vital biological processes such as respiration, photosynthesis and nitrogen fixation. For some of these copper-binding proteins a common fold has been observed, the so-called cupredoxin fold. This fold consists of a rigid Greek key β-sandwich composed of seven or eight parallel and antiparallel β-strands [3], [4], [5]. It can be found in single domain proteins (such as azurin, rusticyanin, amycianin), or as part of multi-domain proteins, such as the copper-containing nitrite reductase (NiR), multi-copper oxidase (MCO, including laccase), or in some cytochrome *c* oxidase subunit. The known physiological role for the majority of copper-binding proteins with a cupredoxin fold is to mediate electron transfer or to catalyze redox reactions [1], [4], [6]. In addition, cupredoxin-like proteins, such as CopC from *Pseudomonas syringae*, have also been proposed to play a role as copper carriers involved in maintaining copper homeostasis [7], [8], [9]. CupA from *Streptococcus pneumoniae* has recently been shown to be associated with Cu^1+^ sequestration, a prerequisite for copper resistance [10]. These recent findings illustrate that despite the fact that cupredoxins have been studied for more than fifty years, novel and intriguing functions are still discovered.

The structure of cupredoxins, the geometry of their copper-binding site, and the electronic configuration of copper have been intensively investigated using a variety of different spectroscopic and structural methods combined with theoretical calculations (for an overview of these methods see [11]).With the exception of the purple Cu_A_-center found in the cytochrome *c* oxidase, which contains two copper atoms, most cupredoxins bind a single copper atom, typically coordinated by one cysteine, one methionine and two histidines (Table S1). Although cupredoxins have these common features, their color can vary from blue to green and red, in the oxidized state (Cu^2+^). This color heterogeneity has been attributed to different geometries of the copper site: from tetrahedral in typical blue copper proteins, to slightly distorted tetrahedral in perturbed-blue copper proteins, distorted tetragonal in green copper proteins and tetragonal in red copper proteins (Table S1) [11]. According to the “coupling distortion” model proposed by Solomon and coll., differences in the bond strength between copper and its ligands as well as different environments surrounding the copper center have been proposed to be the leading cause of these diverse geometries [12]. This model rationalizes the variation of spectroscopic properties of copper sites from blue, to perturbed-blue and to green copper sites (Table S1). This analysis has allowed a classification of cupredoxins into separate subclasses according to their color: type 1 (T1) for blue; type 1.5 (T1.5) for green copper sites; and type 2 (T2) for red copper sites (Table S1). Most of the cupredoxins characterized so far, belong to the T1 subclass. This includes “classic” (azurin, plastocyanin, amicyanin…) [13] and “perturbed” (rusticyanin, cucumber basic protein, pseudoazurin…) blue copper sites (Table S1) [14]. Only a few members of the T1.5 family (e.g., the cupredoxin domain of some NiRs) [15], [16] and only one member of the T2 family (nitrosocyanin) have been characterized in detail so far [17], [18]. During the last decade, several studies demonstrated the possibility to engineer cupredoxins and to obtain variants with different spectroscopic features [19]. For example, by mutating the methionine ligand, a classic blue copper site can be transformed into a green copper site [20], [21], or into a red one [22]. In other cases, it was even possible to change a blue mononuclear-copper site into a purple binuclear-copper site [23], [24]. These results highlight the possibility to transform one copper-binding site into another one, providing novel model representatives to each cupredoxin subfamily. This aids in elucidating the relationship between copper geometry, physicochemical parameters and protein function, such as redox potential, electron transfer efficiency, stability, etc…[13]. A similar contribution will derive from the identification of novel natural cupredoxins from different organisms. We thus took advantage of the existing biodiversity and turned our attention to a novel cupredoxin from the extremophile *Acidithiobacillus ferrooxidans*.

*A. ferrooxidans* is a strict acidophilic, chemolitoautotroph Gram-negative bacterium that optimally grows at pH 2 using iron as an energy source [25]. Since iron is localized outside the cell, this bacterium has developed a “vertical” respiratory chain that spans the outer and inner membranes and involves periplasmic electron transfer carriers (Figure S1) [26], [27], [28]. This vertical respiratory chain has been shown to form a functional supercomplex composed of at least five proteins that couple the oxidation of Fe(II) outside the cells to the reduction of the oxygen at the inner membrane [26]. The couple Fe^3+^/Fe^2+^ has a redox potential of +770 mV vs SHE (Standard Hydrogen Electrode) at low pH. For this reason, this pathway is poorly energetic [29] and requires high redox potential electron transfer carriers. Interestingly, three proteins from this supercomplex possess cupredoxin domains exposed to the acidic environment of the periplasmic compartment: Rusticyanin, a perturbed-blue cupredoxin, CoxB, the subunit of the cytochrome *c* oxidase that contains a purple binuclear-copper center, and AcoP, a cupredoxin associated to the cytochrome *c* and the cytochrome *c* oxidase of *A. ferrooxidans* [25], [30]. While rusticyanin and CoxB homologues have been extensively characterized [31], [32], [33], [34], [35], little is known about AcoP (“Acidophile cytochrome *c o*xidase partner”). It has been shown that AcoP interacts with both cytochrome *c* and cytochrome *c* oxidase, and maintains an optimal cytochrome *c* oxidase activity at physiological pH [30].

In this paper, we conduct an in-depth characterization of the cupredoxin AcoP to answer fundamental questions, regarding the number of copper atoms bound, the putative copper ligands involved, the geometry of the copper site as well as the redox potential. By using a variety of spectroscopic methods, we report intriguing features of AcoP, unique among single-domain cupredoxins. This study emphasizes the importance to identify and fully characterize novel cupredoxins to obtain a more complete picture of cupredoxin's intrinsic properties and functions in “naturally occuring” protein, non-engineered by human.

## Experimental Procedures

### Cloning and expression of acoP

The *acoP* gene was amplified using *A. ferrooxidans* ATCC 23270 genomic DNA as a template. The PCR fragment was inserted into a pDEST17 vector using Gateway™ technology. *Escherichia coli* BL21(DE3) strain was transformed with the resulting plasmid for overexpression. A BIOSTAT Cplus-C10-3 fermentor (Sartorius BBI Systems, Germany), controlled by a micro-DCU system and equipped with pH (405-DPAS-SC-K8S) and pO2 (InPro 6820) sensors (Mettler-Toledo, Switzerland), was used for 10 L batch cultures in LB medium, supplemented with 100 µg/ml Carbenicillin and 1 µM CuSO_4_. The bioreactor was inoculated at OD_600 nm_ of 0.15 with 0.5 L of an overnight flask culture of LB medium and Carbenicillin. The strain was grown aerobically at 1 vvm air, 37°C and pH 7.0 for 2 hours until OD_600 nm_ of 1.0 was reached. To mimic the natural periplasmic environment of *A. ferrooxidans*, the pH of the medium was decreased to 4.9 using a 5N H_3_PO_4_ solution, and maintained at 4.9 over the time courses of the cultivation. Moreover, the temperature was decreased at the same time to 27°C. As soon as O_2_ consumption started (after 45 min and OD_600 nm_ of 1.1), protein expression was induced with 0.1 mM IPTG. After 18 hours of induction, the cells were harvested at 11,000×g and the pellet was frozen at −80°C. The typical yield was in the range of 3.5 g of cells per liter of culture. Several growth conditions were tested to optimize AcoP production in a reproducible manner.

### AcoP Purification

AcoP was purified from the *E. coli* periplasm. The cells were gently resuspended in a Tris/HCl buffer pH 8.0 containing 500 mM sucrose. After 30 min of incubation at 4°C, 10 mM MgCl_2_ and 10 µg/ml DNase from bovine pancreas were added, and the cells were further incubated at room temperature for 30 min. The periplasmic fraction was collected after centrifugation at 7,000×g for 30 min at 4°C and concentrated (JumboSep, Pall Corporation). Subsequent AcoP purification procedure took advantage of AcoP's resistance to low pH when compared to *E. coli* proteins. Successive dialysis steps of the periplasmic extract, using 25 mM glycine pH 2, 0.005% v/v *n*-Dodecyl β-D-Maltoside (DDM), 5% v/v glycerol (Buffer A), followed by another dialysis using 50 mM of sodium acetate pH 5, 0.005% v/v DDM, 5% v/v glycerol (Buffer B) led to the aggregation of the majority of *E. coli* periplasmic proteins. After centrifugation at 13,000×g for 30 min, the supernatant was loaded onto a hydroxylapatite (HTP) column pre-equilibrated in buffer B. Fractions were then eluted using a gradient from 0 to 500 mM of potassium phosphate in buffer B. Fractions were tested for the presence of AcoP by Western Blot, and AcoP-containing fractions were pooled, dialysed and concentrated (Vivaspin, Sartorius). As a final step, the protein was purified using an ion exchange column (MonoS, GE-Healthcare), pre-equilibrated in buffer B. The protein was eluted using a gradient from 0 to 500 mM of sodium chloride in buffer B. Fractions containing purified AcoP were pooled, dialysed, concentrated and analyzed by SDS-PAGE in order to evaluate protein purity (Figure S2). Protein concentration was determined using the Bicinchoninic acid assay (Sigma) with Bovine Serum Albumin as a standard. The addition of 0.005% DDM (below its Critical Micelle Concentration: ∼0.01%) was necessary during the purification steps, presumably to shield hydrophobic regions which could be involved in the interaction of AcoP with its partners: cytochrome *c* oxidase, cytochrome *c* and membranes. Since the concentration of protein samples can also lead to the concentration of DDM, the amount of DDM in the purified sample was estimated. Using Thin Layer Chromatography (TLC), the final amount of DDM was estimated to be under 0.02% (Figure S2B).

### Preparation of holo-AcoP, rusticyanin, and azurin

After the purification, mainly an apoform of AcoP was obtained. To reconstitute the holo protein, purified AcoP was incubated at 4°C in buffer B containing two fold-molar excess of copper. The excess of copper was removed using a gel filtration column (PD10, Pharmacia) and UV-Vis spectra were recorded. Various copper salts were tested under aerobic conditions, including CuCl_2_ or CuSO_4_. Alternatively, apo-AcoP was pre-incubated with CuCl_2_ treated with DL-dithiothreitol (DTT) or was pre-incubated with Tetrakis (acetonitrile) copper (I) hexafluorophosphate in an anaerobic chamber. For each condition, a similar UV-Vis spectrum was obtained. When mentioned, the holoform of AcoP was dialyzed against Universal Buffer (50 mM sodium acetate, 25 mM 2-[N-morpholino]-ethanesulfonic acid (MES), 25 mM 3-[N-morpholino]-propanesulfonic acid (MOPS)) containing 5% glycerol and 0.005% DDM, and adjusted to different pH values (3.5, 5.0 and 7.4).

Recombinant rusticyanin from *E. coli* was purified according to a previously described method [36] and azurin from *Pseudomonas aeruginosa* was purchased from Sigma.

### ICP-MS

Elemental analyses of copper were performed using an ICAP-Q ICP-MS (Thermo Scientific) on purified AcoP, rusticyanin and azurin.

### UV-Visible spectroscopy

Protein samples were fully oxidized using a solution of potassium hexachloroiridate (IV) (K_2_IrCl_6_). Protein concentrations were typically 50 µM for UV-Vis measurements. Room temperature and high temperature UV-Vis spectra were recorded on a Cary 50 Bio (Varian) spectrophotometer equipped with a Peltier-type temperature control system at 293, 303 and 313 K. Low Temperature spectra were obtained with a Cary 300 (Varian) spectrophotometer modified to accommodate a Dewar mounted in the light path. This arrangement allows data collection at around 77 K. For this experiment, samples were diluted in buffer B containing 70% (v/v) glycerol.

### Far-UV and Visible Circular Dichroism (CD) spectroscopy

CD spectra were recorded on a Jasco J-715 spectropolarimeter at 298 K in a 1 mm path length cell. 20 µM of sample diluted into sodium acetate, pH 5 was used. Spectra were averaged from five scans, and normalized for any variation in protein concentrations. Visible-CD spectra were recorded in the region of 300 to 750 nm with 200 µM of the holoform.

### EPR spectroscopy

EPR (Electron Paramagnetic Resonance) spectra were collected at X-band (9.4 GHz) using a Bruker ELEXSYS 500E spectrometer fitted with an Oxford Instruments ESR 900 helium flow cryostat. Simulations of the EPR spectra were performed using the matlab toolbox EasySpin [37].

### UV-Vis redox titration

The midpoint redox potential of the copper center of AcoP was determined by redox titration followed by UV-Vis absorption spectroscopy on a Kontron Instruments UVIKON 932 spectrophotometer. Redox titrations were performed at room temperature in an airtight vessel flushed with oxygen-free argon. Redox potentials were measured with a combined Pt-Ag/AgCl/KCl (3M) micro-electrode calibrated by using redox buffer solutions and are given in the text with respect to the standard hydrogen electrode. The following redox mediators were used at 5 µM final concentrations: 1,1 ferrocene dicarboxylic acid; mono carboxylic acid ferrocene; 1,1′ ferrocene dimethanol; ferrocene; N,N dimethyl-p-phenyldiamine; 1,4-benzoquinone; 1,2-naphtoquinone; phenazine ethosulfate. Reductive and oxidative titrations were carried out by stepwise addition of freshly prepared sodium ascorbate (C_6_H_7_NaO_6_) and potassium hexachloroiridate(IV) (K_2_IrCl_6_) solutions, respectively.

The titration curve A = f(E) was fitted according to the Nernst equation:
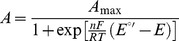
Where A_max_ stands for the absorbance of the fully oxidized solution and E°′ for the standard redox potential of AcoP at pH 5.

## Results

### Bioinformatic analysis of AcoP: a novel cupredoxin

The unusual position of AcoP in a vertical respiratory chain as well as its role in the protection of the cytochrome *c* oxidase led us to investigate the intrinsic properties that could contribute to these functions. A PSI-Blast search, using AcoP sequence as a seed, does not retrieve any cupredoxin of known function or structure. Instead, it retrieves several homologues annotated as either hypothetical proteins or multicopper oxidases (MCOs). Based on e-value, we clustered AcoP homologues from the first PSI-BLAST round into three groups (Figure S3). The first group (identity ≥45%, e-value ≥1^e-46^, coverage ∼100%) includes proteins from acidophiles closely related to *A. ferrooxidans*, such as *A. ferrivorans* and *Thiobacillus prosperus*. These proteins bear a predicted N-terminal transmembrane helix [30]. In the second group, proteins with a lower homology degree (identity ∼35%, e-value from 8^e-20^ to 1^e-13^, coverage ∼70%) are found in other acidophilic organisms, such as *Sulfobacillus acidophilus* and *Sulfobacillus thermosulfidooxidans*. Interestingly, genes encoding for AcoP homologues belonging to these two groups are found to be located in the same gene clusters encoding for cytochrome *c* oxidase. This observation is in line with the proposed role of AcoP in maintaining an optimal cytochrome *c* oxidase activity at acidic pH [30]. More distant homologues (identity ∼30%, e-value ∼1^e-4^, coverage ∼60%; Figure S3, group III) are also found in non-acidophilic, halophilic bacteria and archaea. Although their function is still unknown to our knowledge, most of these AcoP homologues are annotated as putative MCOs, which are multi-domain proteins of about 380 amino acids [38], while AcoP is a single domain cupredoxin of 183 residues. Further rounds of PSI-Blast search, do not help to retrieve any structurally or biochemically characterized proteins, and as such do not provide any further clue about predictable copper site features of AcoP.

**Figure 1:**
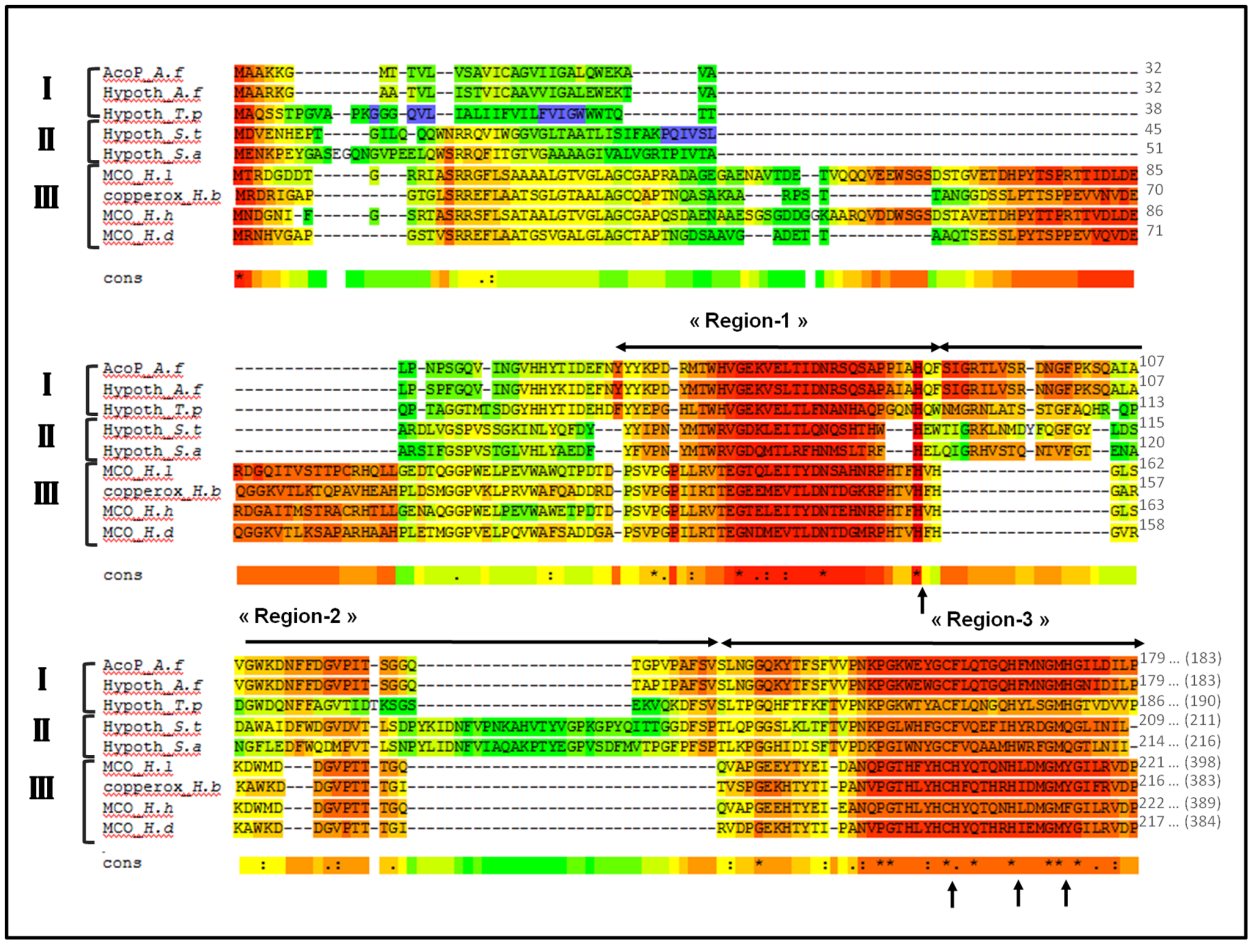
Sequence alignment of AcoP from *Acidithiobacillus ferrooxidans*. The alignment was run on http://www.tcoffee.org/. The extent of conservation is illustrated by a colour scale from blue (bad) to red (good). Conserved amino acids are indicated by (*) for strictly conserved, (:) for highly conserved and (.) for less conserved. I, II and III referred to three separate groups of AcoP homologues that have been clustered based on their e-values. Arrows indicate the putative copper ligands of AcoP (H85, C159, H166 and M171). «Region-1», «Region-2» and «Region-3» are explained in the main text. Only the N-terminal domains of the four last sequences were used. Sequences used for this alignement: AcoP from *Acidithiobacillus ferrooxidans*, NCB Accession: YP_002427513 (AcoP_*A.f*); a hypothetical protein from *Acidithiobacillus ferrivorans*, NCB Accession: YP_004784320.1(Hypoth_*A.f*); a hypothetical protein from *Thiobacillus prosperus*, NCB Accession: ACD03838.1(Hypoth_*T.p*); a hypothetical protein from *Sulfobacillus thermosulfidooxidans*, NCB Accession: WP_020376724.1 (Hypoth_*S.t*); a hypothetical protein from *Sulfobacillus acidophilus*, NCB Accession: YP_004721128.1 (Hypoth_*S.a*); a putative type 3 multicopper oxidase from *Halorubrum lacusprofondi*, NCB Accession: YP_002566552.1 (MCO_*H.l*); a putative copper oxidase from *Halogeometricum borinquense*, NCB Accession: YP_004038099.1 (copperox_*H.b*); a putative type 3 multicopper oxidase from *Halorubrum hochstenium*, NCB Accession: WP_008580876.1 (MCO_*H.h*) and a putative multicopper oxidase from *Haloferax denitrificans*, NCB Accession: WP_004967138.1 (MCO_*H.d*).

Some of these homologues were used for multiple sequence alignment against AcoP using T-Coffee (Figure 1). Overall, we notice a poor level of sequence conservation, apart from two regions, called “region-1” and “region-3”. The latter contains, as in known cupredoxins, three of the four putative copper ligands: C159 X_6_ H166 X_4_ M171. The fourth ligand, usually found in the core of the protein, might be H85 as it is also highly conserved in AcoP homologues (Figure 1). From these *in silico* studies we propose that AcoP and its homologues share a typical cupredoxin copper-binding site [1] (Table S1).

To further investigate possible relationship between AcoP and cupredoxins of known function, we did multiple sequence alignment against cupredoxins of known structure. However, due to the poor level of sequence homology, we couldn't obtain satisfying results. We thus predict the secondary structure of AcoP, using the metaserver PHYRE that combines nine different prediction algorithms [39] (Figure S4A). Based on this prediction, the secondary structure of AcoP is compatible with the β-sandwich fold of cupredoxins, mostly provided by the aforementioned “region 1” (β-sheets 2 to 4) and “region 3”(β-sheets 5 to 7) (Figure 1 and Figure S4B). Further on, known cupredoxin copper-binding residues well match the ones predicted in AcoP (Figure S4B).

Finally, looking at both alignments, we can retrieve some unique features shared only by AcoP and its homologues: (i) a N-terminal transmembrane sequence in group I, replaced by sequences of unknown function in group II and III; (ii) a long “region-2”, not belonging to the cupredoxin fold and not homologous to sequences of known cupredoxins; (iii) in “region-3”, a C-terminal loop of unusual length between the second and fourth copper-binding residues (Figure 1 and Figure S4B). From this analysis, we conclude that AcoP might represent the prototype of a novel, uncharacterized cupredoxin subfamily.

### Heterologous expression of AcoP in E. coli

**Figure 2:**
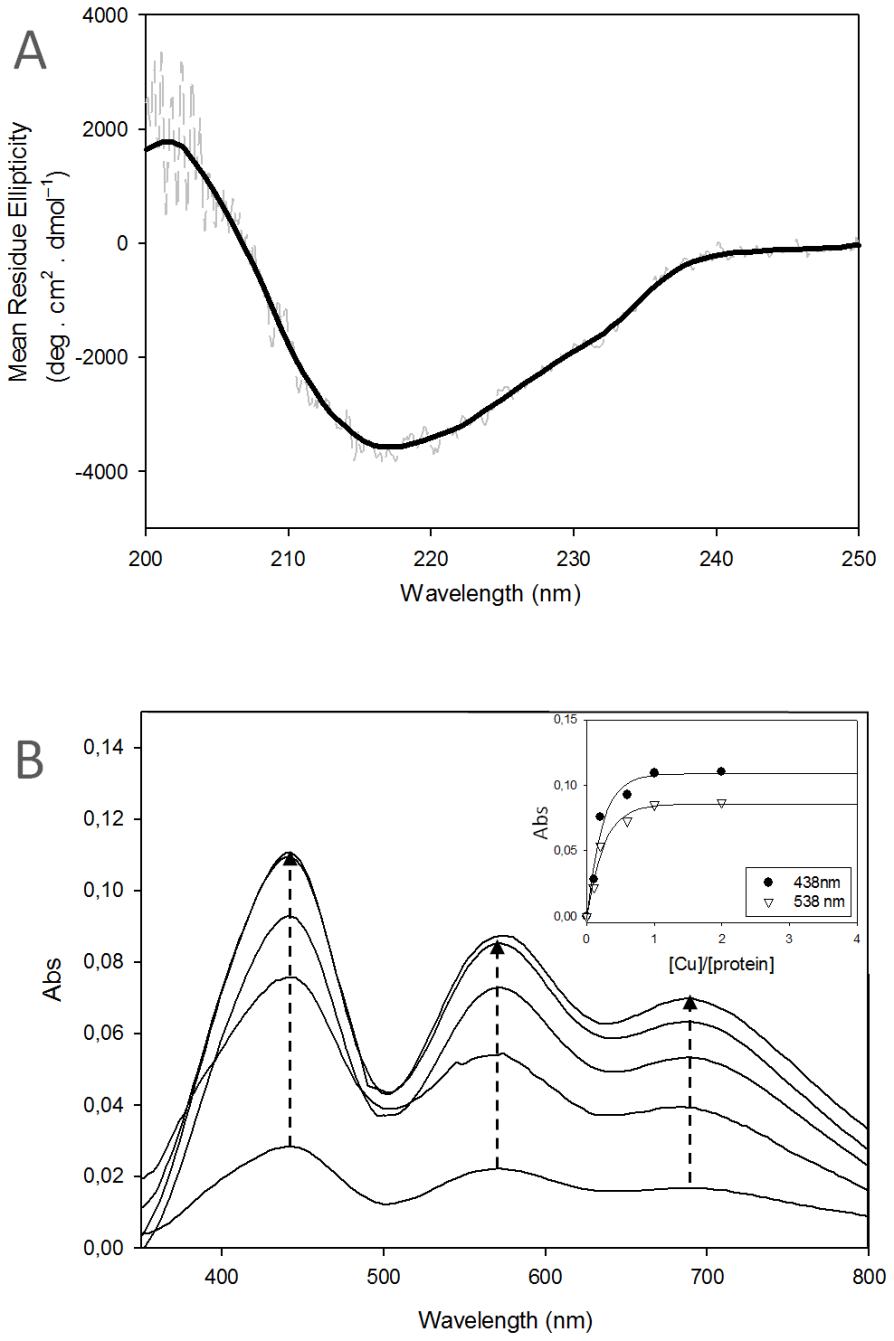
AcoP, a copper protein with a cupredoxin fold. (A) Far-UV CD spectrum of purified AcoP at pH 5, crude data (light grey) and smooth data (dark line). (B) UV-Vis spectra of AcoP after one hour incubation with increasing amounts of copper (CuCl_2_). Ratios of 0.1; 0.2; 0.6; 1; 2 [Cu]/[protein] were used. Arrows show the copper-dependent increment of the absorbance of the three maxima. The spectrum of the apoform was substracted to all spectra. Inset: the absorbance value of the maxima at 438 nm (black triangle) or at 568 nm (white triangle) was plotted versus the ratio of copper. Results presented have been derived from experiments repeated at least three times.

In *A. ferrooxidans* AcoP contains a transmembrane segment and has been shown to be tightly bound to the cytochrome *c* oxidase [30]. This makes studies of endogenous AcoP very challenging, since only low yields of partially purified wild-type AcoP can be obtained from *A. ferrooxidans*. We were however able to purify the periplasmic domain of AcoP from *E. coli* cells grown at low pH (pH 4.6) to mimic *A. ferrooxidans* growth conditions. Analysis of the purified protein by gel filtration, SDS-PAGE, mass spectrometry and N-terminal sequencing reveals a soluble AcoP at high level of purity (Figure S2A and C). To make sure that recombinant AcoP is correctly folded after production in *E. coli*, the structural state of AcoP was monitored by far-UV circular dichroism (CD) spectroscopy (Figure 2A). AcoP's secondary structure content is in agreement with what is expected for cupredoxins, which are rich in β-sheets and display a single minimum around 217 nm. We then tested whether heterologously expressed AcoP maintains its effect toward its partner, the cytochrome *c* oxidase of *A. ferrooxidans* [30]. As shown in Figure S2D, purified AcoP is fully functional in reactivating *in vitro* a population of destabilized cytochrome *c* oxidase.

### Spectroscopic analysis of AcoP, a dark green copper protein

The presence of a putative copper-binding site in AcoP led us to perform spectroscopic analysis of purified AcoP. Like other heterologously expressed cupredoxins [15], [20], purified recombinant AcoP contains a small amount of bound copper, even though *E. coli* cells were grown in copper-supplemented medium. Since it has been shown for other apo-cupredoxins that the addition of exogenous Cu^2+^ allows for their *in vitro* maturation, we added copper (II) and removed the unbound copper by gel filtration. Addition of copper (II) ions to apo-AcoP leads to the apparition of a strong UV-Vis signal within less than one minute of mixing (Figure 2B). Titrations of copper cause a steady increase in the absorption until one equivalent of Cu^2+^ to AcoP was reached (Figure 2B, inset). This result indicates that under these *in vitro* conditions, AcoP binds one copper ion per protein. The same stoichiometry is also confirmed by ICP-MS.

**Figure 3:**
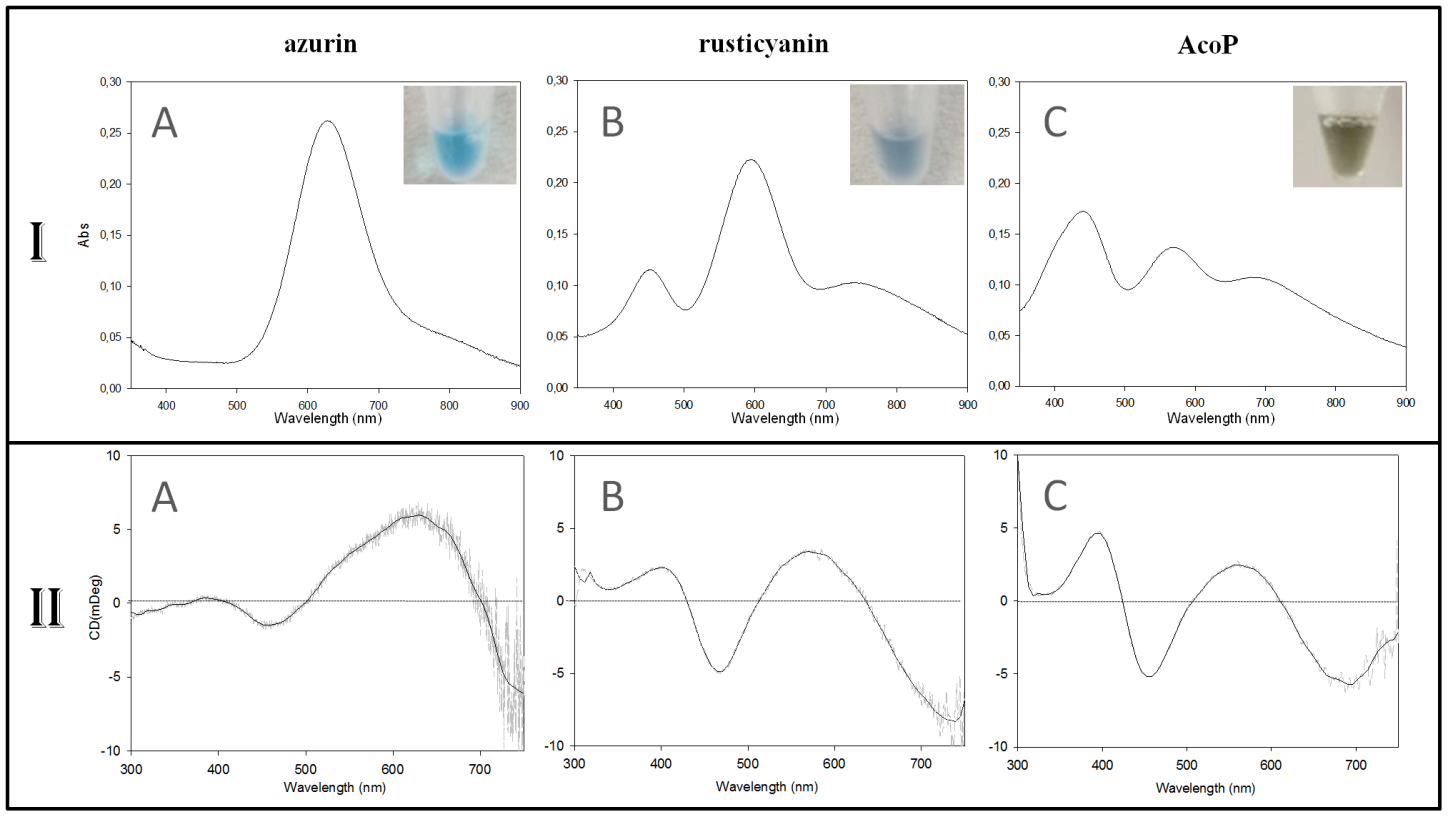
Spectroscopic properties of AcoP in comparison with well-known cupredoxins. (I) UV-Vis and (II) CD spectra of (A) azurin, (B) rusticyanin and (C) AcoP. Both UV-Vis spectra and CD spectra were recorded at pH 5 in buffer B at room temperature. Insets in I: From left to right, color of around 1 mM of azurin (blue), rusticyanin (deep blue) and AcoP (dark green). Both crude data (light grey) and smooth data (dark line) are reported for CD experiment (II). Results presented have been derived from experiments repeated at least three times.

The significant change in the UV-Vis spectrum of AcoP upon copper addition (Figure 2B) is paralleled by the appearance of a dark green color in our sample (Figure 3, inset). To our knowledge, this color has not been reported for any non-engineered single domain cupredoxin. When we compared this spectrum with two well-known single-domain cupredoxins, the “classic” blue copper site-containing azurin from *Pseudomonas aeruginosa*, and the “perturbed” blue copper site-containing rusticyanin from *A. ferrooxidans*, we observed clear differences in their color, indicative of a different copper environment (Figure 3, inset). As expected, azurin exhibits a significant absorption peak around 600 nm responsible for its intense blue color (Figure 3A, Table 1). Several studies established that this band arises from a ligand-to-metal charge-transfer transition between the thiolate sulfur of cysteine and copper (i.e., S(Cys)→Cu π charge transfer transition) that strongly interact with each other [11]. In contrast, rusticyanin exhibits an additional peak at 450 nm (Figure 3B, Table 1), which is also attributed to the interaction between the ligand cysteine and copper, but has been assigned as S(Cys)→Cu σ charge-transfer transition [11]. Indeed, it has been shown that the rotation of the half-occupied Cu (3d_x_^2^_-y_^2^) orbital in perturbed-blue copper sites causes the π interaction to weaken, while σ interactions achieve better overlap and gain intensity [11]. This rotation is associated with a decrease of the methionine-to-copper bond length and an increase of the cysteine-to-copper bond length, while causing a change in copper geometry from a tetrahedral to a distorted tetrahedral geometry (Table S1). The absorption spectrum of AcoP is dominated by an intense band at 438 nm (Figure 3C, Table 1), compatible with a S(Cys)→Cu σ charge-transfer transition, as in perturbed-blue and green copper sites. In addition, AcoP exhibits a weaker S(Cys)→Cu π charge transfer transition at 568 nm and a broad peak around 690 nm which likely correspond to d-d transition bands (Figure 3C and Table 1) [11]. Treatment with ascorbate reduced Cu^2+^-AcoP and resulted in the complete and simultaneous bleaching of all three peaks. In oxidized cupredoxins, the ratio between absorbance at the ∼450 and ∼600 nm peaks is defined in the literature as the R_L_ value and correlates to the extent of tetragonal distortion [22]. The R_L_ value is used to classify cupredoxin copper centers into type 1 (R_L_<1), type 1.5 (1<R_L_<2) or type 2 (R_L_>2) (Table S1). We find an R_L_ value of 1.3 for AcoP (Table 1), that can be assigned to the type 1.5 category. This result clearly suggests that the copper site in AcoP belongs to neither the blue nor the perturbed-blue class (type 1). Noteworthy, an R_L_ value of 1.3, as in AcoP, was rarely found among members of the large cupredoxin family (Table S1). Spectra of oxidized holo-AcoP were collected at different pH values (from 3.5 to 7.4) (Figure S5). We did not observe significant changes, indicating that the unusual spectroscopic features of AcoP are not due to low pH, and that copper geometry is stably preserved on a wide pH range.

**Table 1:** Spectroscopic and EPR Spin parameters for azurin, rusticyanin and AcoP.

	UV-Visible spectroscopy			CD spectroscopy	EPR spectroscopy	
	S(Cys)→Cu σ charge transfer transition	S(Cys)→Cu π charge transfer transition	R_L_ (≈450 nm/≈600 nm)	CD extrema bands	g values	A values [×10^−4^ cm^−1^]
				390(+) nm	g_x_ = 2.032	A^Cu^_x_ = 7
**azurin**	-	627 nm	0.08	460(−) nm	g_y_ = 2.056	A^Cu^_y_ = 7
				640(+) nm	g_z_ = 2.259	A^Cu^_z_ = 58
				402(+) nm	g_x_ = 2.020	A^Cu^_x_ = 64
**rusticyanin**	450 nm	597 nm	0.4	471(−) nm	g_y_ = 2.062	A^Cu^_y_ = 9
				577(+) nm	g_z_ = 2.217	A^Cu^_z_ = 56
				400(+) nm	g_x_ = 2.019	A^Cu^_x_ = 65
**AcoP**	438 nm	568 nm	1.3	460(−) nm	g_y_ = 2.057	A^Cu^_y_ = 12
				565(+) nm	g_z_ = 2.193	A^Cu^_z_ = 66

To further characterize the copper center environment of AcoP, CD spectra in the visible range were recorded (Figure 3). We find that the AcoP spectrum displays a significant increase of intensity at 400 nm over 565 nm, when compare to the rusticyanin and azurin spectra (Figure 3 and Table 1). Previous studies on cupredoxins showed that CD extrema peaks, as for the visible absorption peaks, can be assigned to the ligand-to-metal charge transfer transitions [40]. These results are in agreement with UV-Vis data, and suggest that the copper site in AcoP is different from blue and perturbed-blue copper sites, and similar to green ones [16].

**Figure 4:**
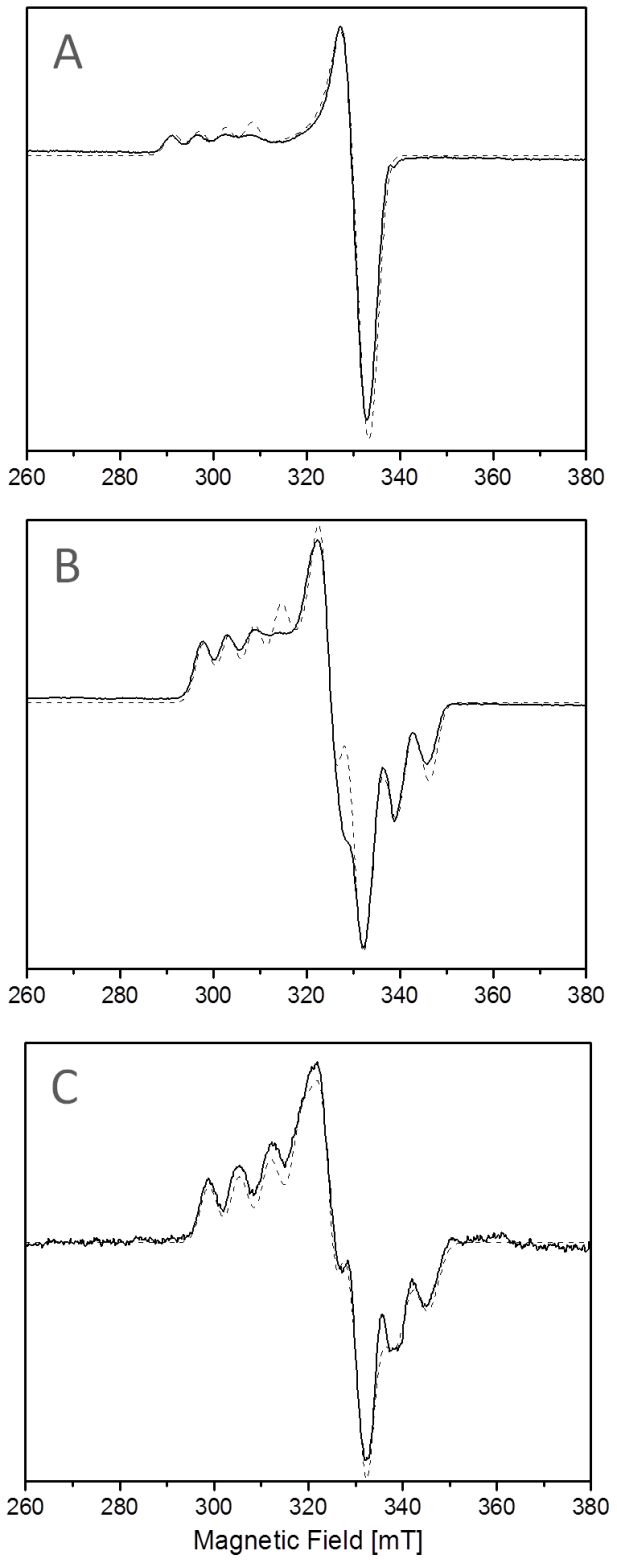
Comparative EPR study of cupredoxins. X-band EPR spectra of (A) azurin, (B) rusticyanin and (C) AcoP at pH 5 in buffer B at 15 K. The EPR spectra are in solid line while the simulated spectra are in dashed line. Results presented have been derived from experiments repeated at least three times.

**Figure 5:**
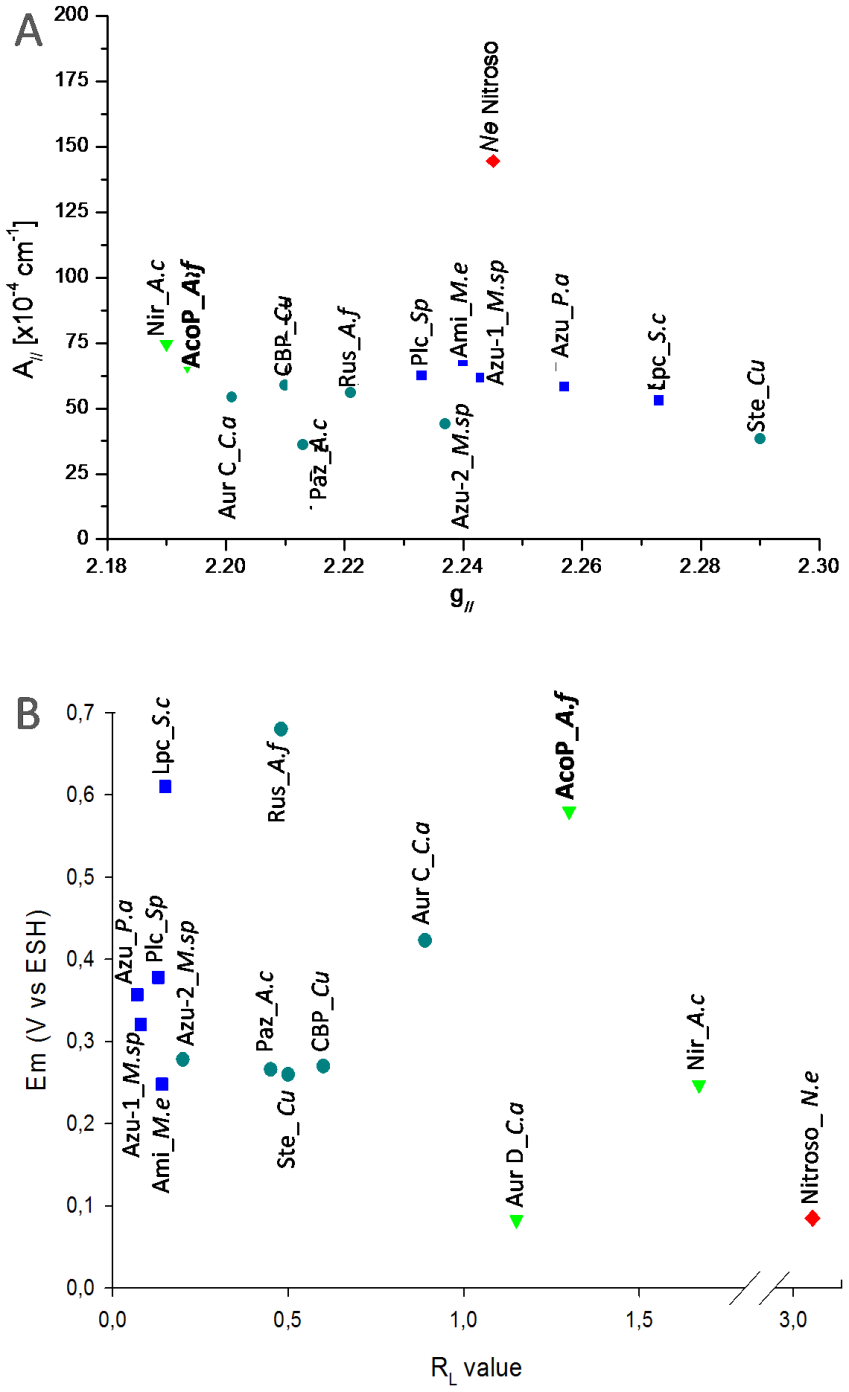
AcoP intrinsic properties compared with several blue (dark blue squares), perturbed-blue (light blue circles), green (green triangles) and red copper (red diamonds) sites. (A) Peisach-Blumberg diagram correlation between A_//_ versus the g_//_ EPR parameters [43] and (B) correlation diagram between the redox potential (Em) and the R_L_ value. Color codes used for these diagrams are based on spectroscopic features (see Table S1) rather than reported visual colors. Abbreviations used: AcoP*_A.f*: Acidophile cytochrome *c* oxidase Partner from *Acidithiobacillus ferrooxidans* (current study); Ami*_M.e*: amicyanin from *Methylobacterium extorquens* [59]; Aur C*_C.a* and Aur D*_C.a*: auracyanin C and D from *Chloroflexus aurantiacus* [48]; Azu-1_ *M.sp* and Azu-2_ *M.sp:* azurin isoform 1 and 2 from *Methylomonas sp.* [59]; Azu_*P.a:* azurin from *Pseudomonas aeruginosa* [55], [60]; CBP_*Cu:* cucumber basic protein from *Cucumber* [19]; Lpc_*S.c:* lipocyanin from *Streptomyces coelicolor* [61]; Nir_*A.c:* Nitrite reductase from *Achromobacter cycloclastes* [19]; Nitroso_*N.e:* nitrosocyanin from *Nitrosomonas europaea* [62]; Paz_*Ac*: pseudoazurin from *Achromobacter cycloclastes* [60]; Plc_*Sp*: plastocyanin from *spinach* [60]; Rus*_ A.f:* rusticyanin from *Acidithiobacillus ferrooxidans* (current study) [34]; Ste*_Cu:* stellacyanin from *Cucumber* [63], [64].

To get further insight into the copper site geometry of AcoP, we performed EPR experiments. EPR spectroscopy is commonly used to study the ground state wave function of copper sites. With this technique, we can observe characteristic Cu^2+^ small hyperfine splitting constant (A_//_) (Table S1). Further on, the interaction between the unpaired electron spin and the nuclear moment of the Cu^2+^ ion (A_//_ EPR parameter) is an accurate indicator of the electron delocalization onto the thiolate, and thus of the copper site covalency in the electronic ground state [12]. Consequently, EPR spectroscopy allows to distinguish between classic blue copper sites (axial EPR signature) and perturbed-blue or green copper sites (rhombic EPR signature) (Table S1) [19], [41], [42]. Figure 4 shows frozen solution EPR spectra of the typical axial azurin (A), of the rhombic rusticyanin (B) and of AcoP (C). The EPR spectrum of oxidized AcoP displays rhombic features with three well-distinguishable g-values. By using the following EPR-parameters: g_x_ = 2.019; g_y_ = 2.057; g_z_ = 2.193 and A^Cu^_x_ = 65×10^−4^, A^Cu^_y_ = 12×10^−4^ and A^Cu^_z_ = 66×10^−4^ cm^−1^ (A^Cu^_z_, also called A_//_), we obtained excellent agreement between the experimental data and our simulations. As for the optical spectra, the EPR spectra of AcoP do not show significant changes with pH from 2 to 7 (Figure S5), meaning that the overall geometry is not modified under these conditions. The AcoP EPR parameters (Table 1) are similar to those published for a cupredoxin domain of *Achromobacter cycloclastes* nitrite reductase NiR [19], a natural cupredoxin with a strongly perturbed copper site, causing the green color. By plotting AcoP into a well-known Peisach-Blumberg diagram which correlates g_//_ and A_//_ parameters for mononuclear copper sites, we indeed observe that the EPR signature of AcoP correlates well with that of the green copper site (Figure 5A) [43]. These EPR results are also consistent with optical spectroscopic data of AcoP (R_L_ = 1.3), and indicative of a potential distorted geometry of its copper center, similar to the one observed for the green NiR domain from *A. cycloclastes*.

### A constrained copper site with temperature-independent behavior

**Figure 6:**
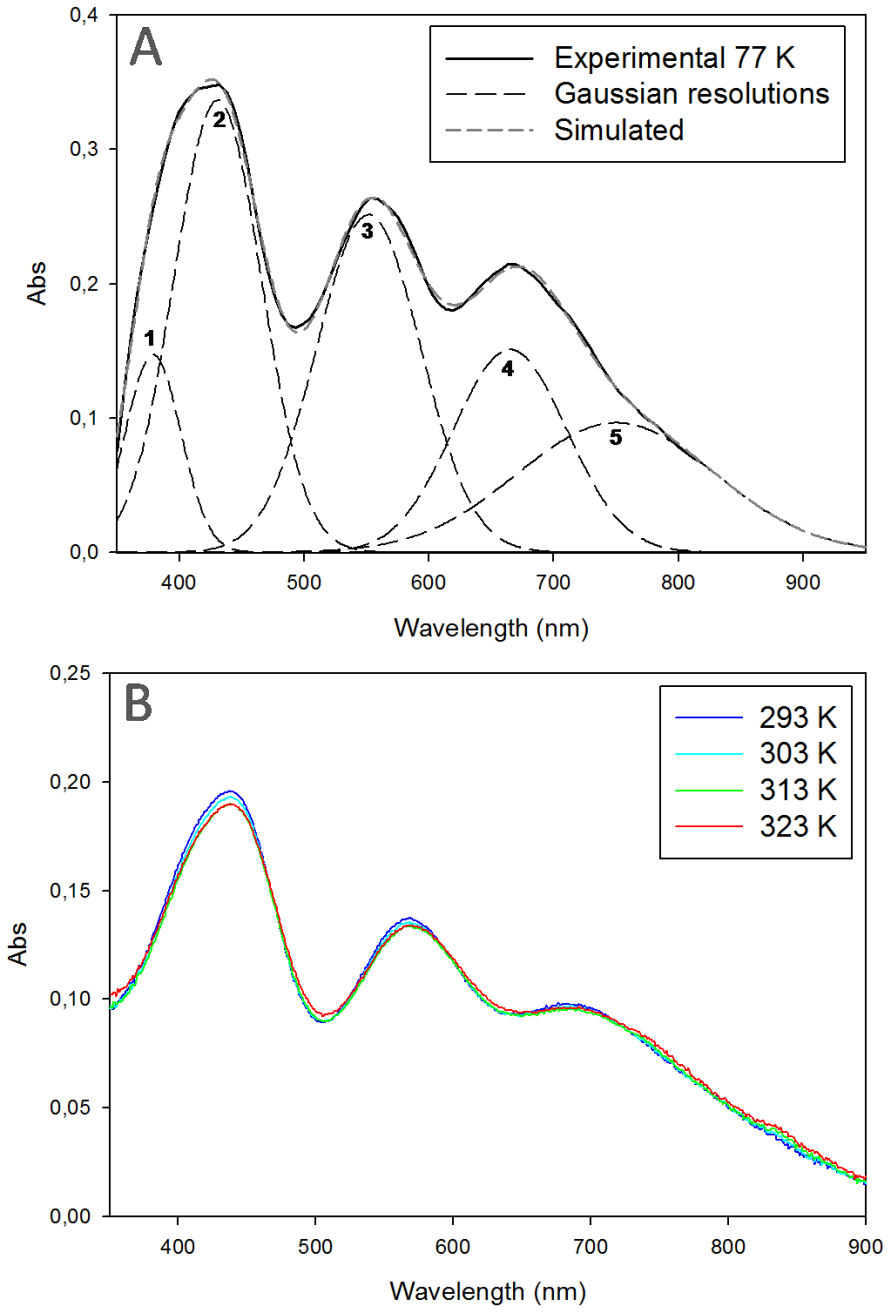
AcoP spectroscopic behavior upon temperature variation. (A) Low temperature UV-Vis spectrum of AcoP at 77 K at pH 5 in buffer B containing 70% (v/v) glycerol (Black solid line). Gaussian resolutions (Black dashed line) have been obtained from a simultaneous fit of the absorption spectrum. Maxima from Gaussian 1 to Gaussian 5 are 375 nm, 425 nm, 545 nm, 660 nm, 745 nm respectively. The resulting simulated spectrum is illustrated as grey dashed line. (B) UV-Vis spectra of AcoP at pH 5 in buffer B at various temperatures: 293 K (dark blue); 303 K (light blue); 313 K (green) and 323 K (red). Results presented have been derived from experiments repeated at least three times.

Low temperature UV-Vis spectroscopy is often used to get a more defined spectrum of a species with an increase in absorption bands and a reduction of their half-band width. The low temperature spectrum of AcoP is shown in Figure 6A. The Gaussian resolutions, obtained from a simultaneous fit of the low temperature absorption spectrum of AcoP, are included in Figure 6A. Upon comparison of the spectra with data obtained from *A. cycloclastes* NiR [16], we conclude that the band, which is observed at 375 nm likely corresponds to a Cu-S(Met) charge transfer transition, while the bands observed at 425 and 545 nm corresponds to a S(Cys)→Cu σ and a S(Cys)→Cu π charge-transfer transition, respectively. The two bands around 650 to 750 nm are usually assigned to d-d transition bands.

As shown in Figure 6A and B, the spectral behavior of AcoP is not affected by temperature variations and the relative ratio between the two main absorption bands ∼450 nm and ∼600 nm remains unchanged over the tested temperature range of 77 K to 323 K (Figure 6). This is in contrast with previous studies on the green copper site of NiR, whose absorption spectrum showed a significant temperature dependence [44]. At low temperature, the NiR site was shown to have an intense σ charge transfer band at 460 nm. After raising the temperature, the intensities of the bands at 570 and 460 nm increased and decreased, respectively. This large temperature dependence of NiR has been attributed to an unconstrained copper site [44], and it explains NiR spectral features at room temperature, with a mix of green and blue copper species. Our results suggest that, in contrast to what we observe with NiR, AcoP copper center has a single species at all temperatures. This case is similar to that of proteins, such as plastocyanin and azurin, which also have a higher degree of constraint, if compared to the green domain of *R. sphaeroides* NiR [45].

### AcoP has the highest known redox potential among green cupredoxin

Even though all single domain cupredoxins have a very similar fold, their redox potentials greatly vary between +85 to +680 mV with the majority of their redox potentials ranging from +120 to +370 mV (Table S1) [46]. These redox potentials are often significantly higher than the redox potential of the aqueous Cu^2+^/Cu^1+^ couple (+160 mV), pointing towards a stabilization of the reduced *versus* the oxidized state of the copper ion by the protein.

**Figure 7:**
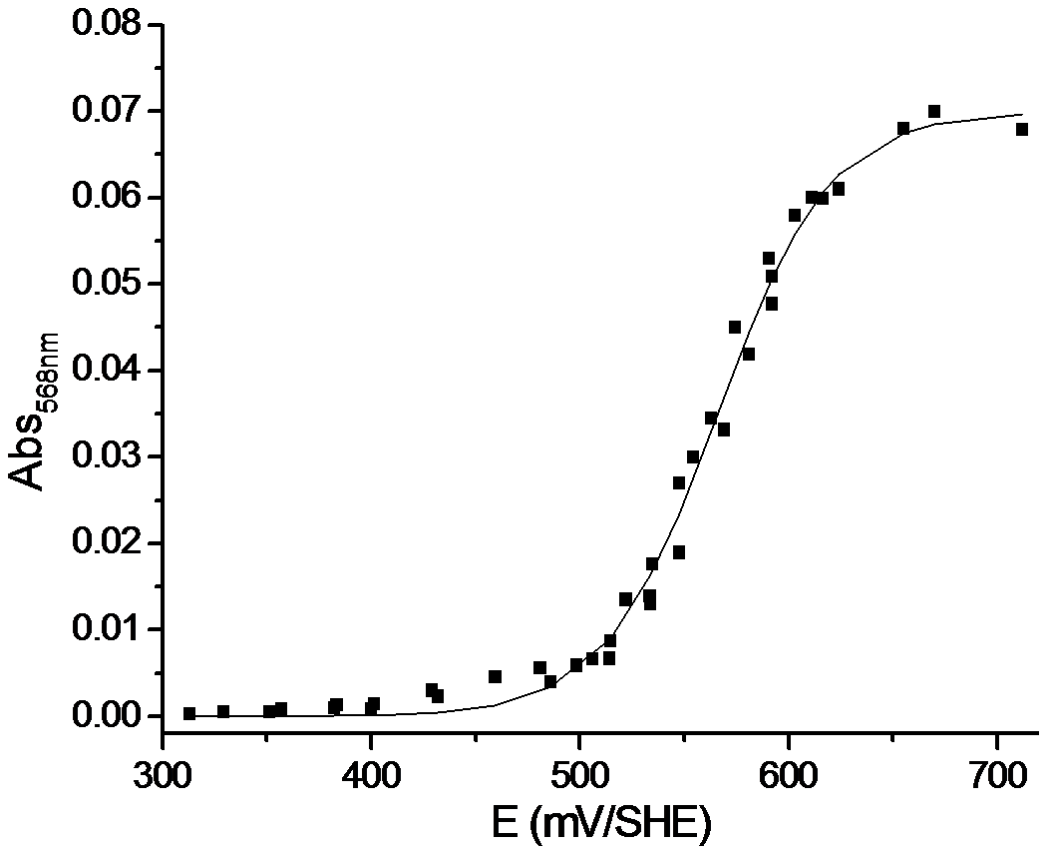
Determination of AcoP redox potential. Redox titration of AcoP at pH(K_2_IrCl_6_) or reductant (C_6_H_7_NaO_6_) were used to oxidize or reduce the sample. The absorbance at 568 nm was plotted versus the redox potential of the solution. AcoP reduced spectrum was used to normalized the datas.

To determine the redox potential of AcoP, the protein was stepwise oxidized with hexachloroiridate or reduced with ascorbate. We monitored the redox potential of the solution, and, in parallel, recorded the UV-Vis spectrum of the protein in order to determine the maximum absorption of AcoP at 568 nm (Figure 7). After fitting the data for a one-electron reaction (*n = 0.94*) using the Nernst equation, we obtain a midpoint potential of +566 mV at pH 5. This redox potential is significantly higher than the redox potentials determined for any other known green copper sites (Figure 5B).

## Discussion

In this work we report the biophysical characterization of recombinant AcoP, a novel member of the cupredoxin family. We propose that AcoP constitutes the first member of a novel subfamily of green cupredoxins, based on a number of very unique properties: (i) it is the first single domain cupredoxin found to have a constrained green copper site; (ii) it contains an unusually long C-terminal loop between the second and the fourth copper ligands (Cys and Met, respectively); (iii) it has the highest redox potential reported to date for a green-type cupredoxin; and (iv) it is the first green cupredoxin involved in aerobic respiration.

### Discovery of a single domain cupredoxin with a green copper site

The green color of purified holo-AcoP together with its spectroscopic parameters, were highly reminiscent of NiR green domain [16], [47]. This was an unexpected result since, until now, no single domain cupredoxin found in nature had been shown to be green, nor to be involved in an oxygen reduction pathway. While several blue cupredoxins have been spectroscopically and functionally characterized, only a few natural members from the green and red color families have been studied so far. Very recently, two other single domain cupredoxins of unknown functions have been found in *Chloroflexus aurantiacus*: auracyanin C and D [48]. They have unusual colors (grey and light green, respectively), spectra and R_L_ values. This finding, together with our results, provides evidence that the list of cupredoxins is far from complete. Thus, although cupredoxins have long been the subject of thorough biochemical and biophysical studies, novel properties are still discovered for this intriguing family of proteins.

The structural features that account for green color in copper-binding proteins have been extensively studied for the green domain of NiR from *R. sphaeroides* and *A. cycloclaste* as well as for engineered cupredoxin that has spectroscopic features strongly similar to AcoP such as Azurin Met121Cys, Met121His and Met121Glu mutants [22]. When compared to blue copper sites, green copper sites show major structural differences, including a shorter Cu-S(Met) bond length, a longer Cu-S(Cys) bond length, and an overall distortion of the Cu site geometry from tetrahedral to distorded tetragonal (Table S1) [16], [49]. By analogy, we propose that AcoP's green copper site is also tetragonally distorted. It is unclear, yet, whether this geometry variation is due to changes in bond lengths or due to the second sphere of coordination that could also modify the copper surrounding. What is clear, however, is that evolution has selected a single domain cupredoxin with classical copper ligands but with a copper center geometry yet rarely found in nature. Together with the finding of a large number of AcoP homologues, these results point to a more widespread role for green cupredoxins in respiratory chains than previously expected.

### Determinants for a high redox potential

Several studies have attempted to explain the wide range of redox potential values in single-domain cupredoxins [46], [50]. Site directed mutagenesis has shown that redox potential can be modulated by amino acid substitution in the vicinity of copper [51], [52], [53]. As observed for rusticyanin, hydrophobic patches close to the metal binding site appear to increase redox potentials, since they exclude water or residues with electronegative ligand atoms [54], [55].

A change of the axial ligand can also influence the redox potential. Indeed, the natural replacement of the axial methionine by a hydrophobic residue, like leucine, has been shown to increase the redox potential in cerruloplasmin [56]. Similarly, replacement of the axial methionine with threonine turns the green NiR of *R. sphaeroides* into a blue protein, weakens copper interaction with the axial ligand, and causes a 100 mV increase in the redox potential [15].

By plotting the R_L_ value versus the redox potential of well known cupredoxins (Figure 5B), we can observe that all naturally-occuring cupredoxins are characterized by a low redox potential when the R_L_ value exceeds 1. A similar trend is also observed for Azurin Met121 mutants, that include green and red variants [22]. On the other hand, AcoP appears like an exception to this general trend. As such, the present study emphasizes that metal coordination as well as copper center geometry are not the only determinants for the redox potential.

### AcoP sequence analysis, the potential determinants for a green geometry coupled with a high redox potential?

One unresolved question, however, concerns the structural aspects of the AcoP copper site that imparts its unusually high redox potential and its green color. Bioinformatics analysis points to unique features of AcoP and its close homologues that could lead to such unusual properties: (i) the presence of a region of unknown function, conserved among AcoP-like proteins and absent in other cupredoxins (“region-2” in Fig. 1); (ii) a few relatively conserved residues (“region-3” in Figure 1) might also act on the second sphere of coordination in AcoP homologues (Q162, T163, M168, H172); (iii) within the C-terminal loop, an unusual distance of six residues between C159 and H166 (to our knowledge, never reported to date). Usually, these cysteine and histidine are separated by seven-to-eight amino acids in green NiRs, and two-to-four in blue and red cupredoxins [1]. This loop is particularly well known to play an important role in cupredoxins, as it can affect the second sphere of coordination due to different lengths, primary sequence and conformations. The effect of changing the length of this loop in cupredoxins has been intensively studied during these past years [57]. Dennison and coll. have proposed that the loop length rather than its sequence dictates the geometry of the copper center [58]. In AcoP, the C-terminal loop length may account for a more constrained copper center than in NiR, characterized by a longer loop and a pretty unconstrained copper site [44]. Further structural analysis will be required to determine the determinants of these unusual properties.

### Involvement of three cupredoxins with different copper geometries in the same pathway

The iron-oxidizing respiratory supercomplex from *A. ferrooxidans* contains three distinct cupredoxins: Rusticyanin, CoxB and AcoP, containing a perturbed-blue, a purple and an unusual green copper site, respectively (Figure S1). This suggests that each of these cupredoxins might play a specific role that requires a different copper geometry. Rusticyanin function has been well established as an electron shuttle between the outer membrane cytochrome Cyc2 and the periplasmic cytochrome *c*. CoxB, with its binuclear copper center, is a subunit of cytochrome *c* oxidase and the entry point for electrons [35]. Concerning AcoP, we previously showed that it has an unprecedented dual interaction with two well-known redox partners: cytochrome *c* and cytochrome *c* oxidase. Associated with the latter, AcoP has been shown to be responsible for its optimal activity [30]. Based on the finding that AcoP has a high redox potential, an additional role in electron transfer could also be envisaged.

Green copper centers have yet been rarely found in nature. This study highlights the existence of a novel kind of green copper centers in single domain cupredoxins involved in respiratory pathways. This finding emphasizes the significance of relaying on biodiversity to uncover novel features of supposedly well-characterized protein families. Future studies on the three distinct cupredoxins found in the same respiratory pathway will help decipher the determinants of their specificity and their respective mode of action.

## Supporting Information

Figure S1 **Model of the ferrous iron oxidation pathway of** ***Acidithiobacillus ferrooxidans*** **.** This respiratory chain couples the oxidation of Fe^2+^ to Fe^3+^ (at the outer membrane) with the reduction of oxygen to water (at the cytosolic side of the inner membrane). This chain is composed of the cytochrome Cyc2, anchored, to the outer membrane and responsible for Fe^2+^ oxidation (*light salmon*), the periplasmic blue copper protein Rusticyanin (*blue*; PDB # 1RCY), the diheme cytochrome *c* (*orange*; PDB # 1H1O), the green copper protein AcoP (*dark green*; model obtained with a low level of confidence and based on PDB # 2BWI) and an integral inner-membrane *aa_3_*-type cytochrome *c* oxidase (the four subunits, Cox A, B, C and D are in *shades of green*; models based on PDB # 1QLE). Solid and dashed arrows indicate the proposed model for electron (e^−^) and proton (H^+^) transfer pathways, respectively. This scheme is adapted from Roger et *al.* [28].
(TIF)

Figure S2 **Analytical procedures.** (A) Protein purity. Coomassie blue staining of purified azurin (*lane 1*), rusticyanin (*lane 2*) and AcoP from *E. coli* (*lane 4*) run on 15% SDS-PAGE. 15 µg of proteins were loaded on the gel. Molecular mass markers are indicated in *lane 3*. (B) Thin layer chromatography of purified AcoP. 5 µL of sample was loaded (*lane a*). We can estimate that the DDM amount in AcoP sample is very low (less than 0.02%), by direct comparison with increasing quantities of DDM (1, 2, 5 10 and 20 µg, lane b to f). (C) Gel filtration of purified AcoP. 3 mg of sample was loaded on S 75 gel filtration column using an ÄKTA basic FPLC setup. A single major molecular peak on the chromatogram was obtained. Fractions from this peak gave a single band on SDS-PAGE. (D) Effect of recombinant AcoP on the cytochrome *c* oxidase (C*c*O) activity from *A. ferrooxidans*. The relative 100% corresponds to the activity of a partially destabilized cytochrome *c* oxidase from which the specific activity is 0.3 µmol/mg/min. AcoP alone does not present any cytochrome *c* oxidase activity. The addition of recombinant, reconstituted holo-AcoP (black bar) has a positive effect on the cytochrome *c* oxidase activity compared to the addition of equivalent amounts of buffer (dark grey bar). Results presented correspond to an average of three experiments.
(TIF)

Figure S3 **Distribution of sequence coverage and identity for 28 AcoP homologues.** PSI-Blast was run on http://blast.ncbi.nlm.nih.gov using the AcoP sequence as a template (NCB Accession: YP_002427513). Dark and light grey bars represent the percentage of sequence coverage and identity respectively. Group (I) and (II) include hypothetical proteins from acidophiles with two different scores for sequence coverage and identity (e-values from 3^e-132^ to 1^e-46^ and from 8^e-20^ to 1^e-13^, respectively); (III) includes N-terminal domains of putative copper and multicopper oxidases (e-values from 1^e-4^ to 1^e-3^). (*) correspond to sequences used for multiple sequence alignment (see Figure 1). Abbreviations used: Hypoth: hypothetical protein; MCO: multicopper oxidase; copperox: copper oxidase; Cu-Nir: Nitrite reductase copper containing protein.
(TIF)

Figure S4 **Sequence alignement based on secondary structure elements of AcoP with cupredoxins of known 3D structure.** (A) Secondary structure prediction of AcoP using PHYRE [39]. Alpha helices (h), beta sheets (e) and random coiled (c) are colored in red, blue and grey, respectively. Two bottom lines: consensus sequence and consensus probability (“cons_prob”); 9 (red) high prediction probability; 2 (cyan) the lowest probability in this prediction. A metaserver approach was required for this secondary structure prediction because we obtained very different result using single software analysis. Accordingly, a very limited set of secondary structure elements has been predicted with high confidence. (B) Sequence alignement of secondary structure elements of AcoP (predicted using PHYRE, see A) with well-known cupredoxins. Secondary elements of the cupredoxin fold are reported on top. Yellow, light green, cyan, black, dark green, magenta and red arrows: β-Strand 1 to 7. The C-terminal a-helical loop (C-ter loop), between the second and fourth copper ligand, is also reported (grey cylinder). Vertical black arrows indicate copper binding ligands. Aligned sequence: azurin from *Pseudomonas aeruginosa* (Azu_*P.a*), PDB # 1E65; plastocyanin from *Populus nigra* (Plc_*P.n*), PDB # 1PLC; pseudoazurin from *Achromobacter cycloclastes* (Paz*_A.c*), PDB # 1ZIA; rusticyanin from *Acidithiobacillus ferrooxidans* (Ru*s_A.f*), PDB # 2CAK are well studied single-domain blue copper proteins with known structure. Nitrite reductase from *Achromobacter cycloclastes* (Nir*_A.c*), PDB # 2BWI is a model of a green copper center and it belongs to the multi-domain cupredoxin subfamily. Nitrosocyanin from *Nitrosomonas europaea* (Nitro_*N.e*), PDB # 1IC0A belongs to the red cupredoxin subfamily.
(TIF)

Figure S5 **pH-independent spectroscopic behavior of AcoP.** (A) UV-Visible spectra of AcoP (50 µM) in universal buffer (50 mM sodium acetate, 25 mM MOPS, 25 mM MES) at pH 3.5 (solid line); pH 5.0 (dashed-line) and pH 7.4 (dotted-line). (B) Spectroscopic parameters of AcoP at pH 3.5, pH 5.0, and pH 7.4. (*)Values obtained in the universal buffer pH 5 show a slight shift (2 nm) if compared to the ones from buffer B pH 5 (Table 1). (C) Frozen solution X-band EPR spectra of AcoP at pH 2.0, 5.0 and 7.0. Experimental conditions: T = 15 K, microwave frequency 9.48 GHz, modulation amplitude 2 mT, microwave power 1 mW.
(TIF)

Table S1 **Overview of structural and spectroscopic properties of some “Blue”, Green” and “Red” copper sites.** (*) refers to the axial ligand. The axial residue is usually a methionine, however, some T1-copper site (such as stellacyanin) can have a glutamine residue as axial ligand [59].
(TIF)
