# Supplementary figures and images for: Spectroscopic Characterization of a Green Copper Site in a Single-Domain Cupredoxin
Source: PLoS One. 2014 Jun 16;9(6):e98941. doi: 10.1371/journal.pone.0098941 (PMC4059628; doi:10.1371/journal.pone.0098941)

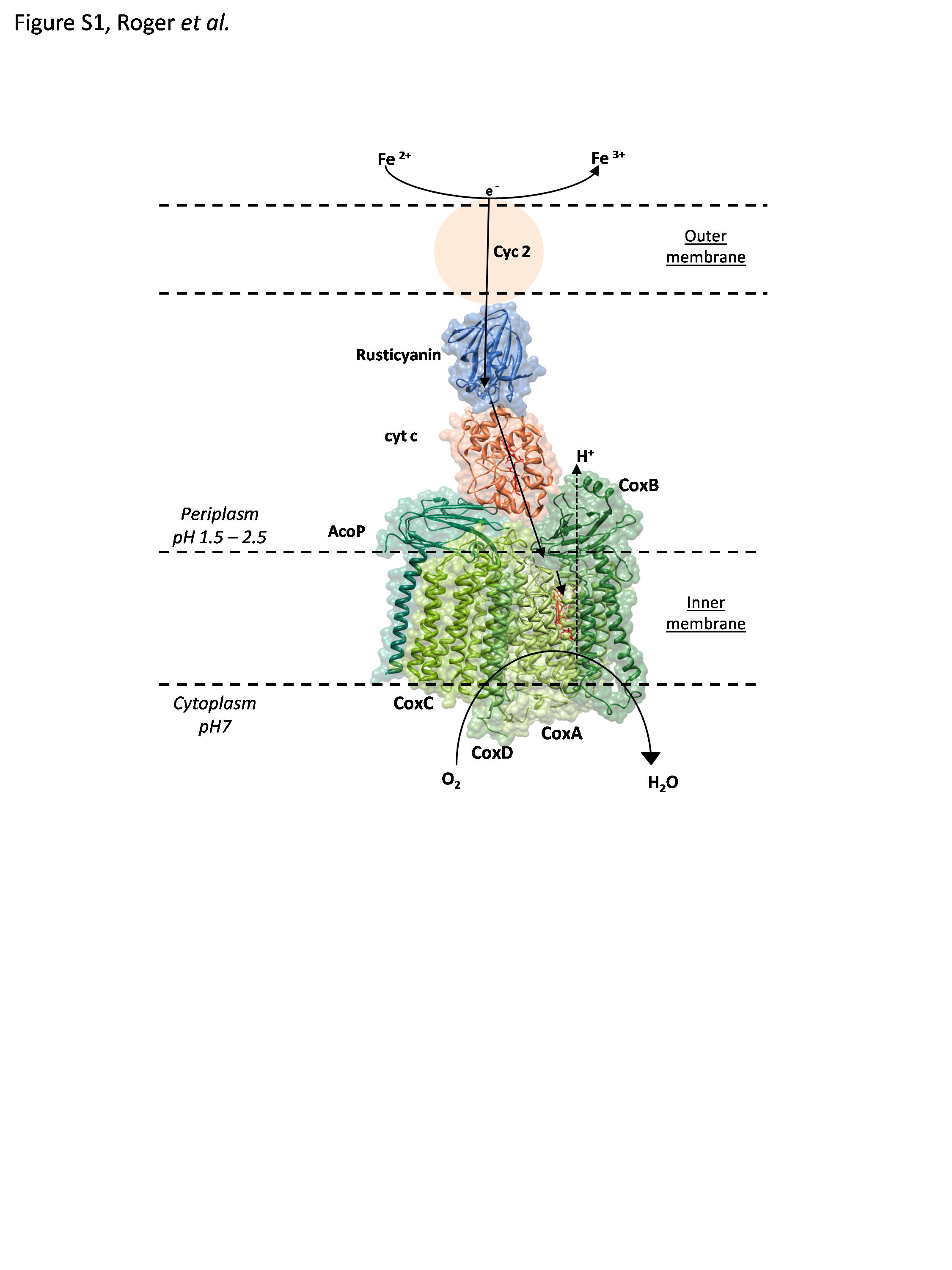

Supplement: Figure S1 — Model of the ferrous iron oxidation pathway of Acidithiobacillus ferrooxidans . This respiratory chain couples the oxidation of Fe2+ to Fe3+ (at the outer membrane) with the reduction of oxygen to water (at the cytosolic side of the inner membrane). This chain is composed of the cytochrome Cyc2, anchored, to the outer membrane and responsible for Fe2+ oxidation (light salmon), the periplasmic blue copper protein Rusticyanin (blue; PDB # 1RCY), the diheme cytochrome c (orange; PDB # 1H1O), the green copper protein AcoP (dark green; model obtained with a low level of confidence and based on PDB # 2BWI) and an integral inner-membrane aa3-type cytochrome c oxidase (the four subunits, Cox A, B, C and D are in shades of green; models based on PDB # 1QLE). Solid and dashed arrows indicate the proposed model for electron (e−) and proton (H+) transfer pathways, respectively. This scheme is adapted from Roger et al. [28]. (TIF) [file pone.0098941.s001.tif]

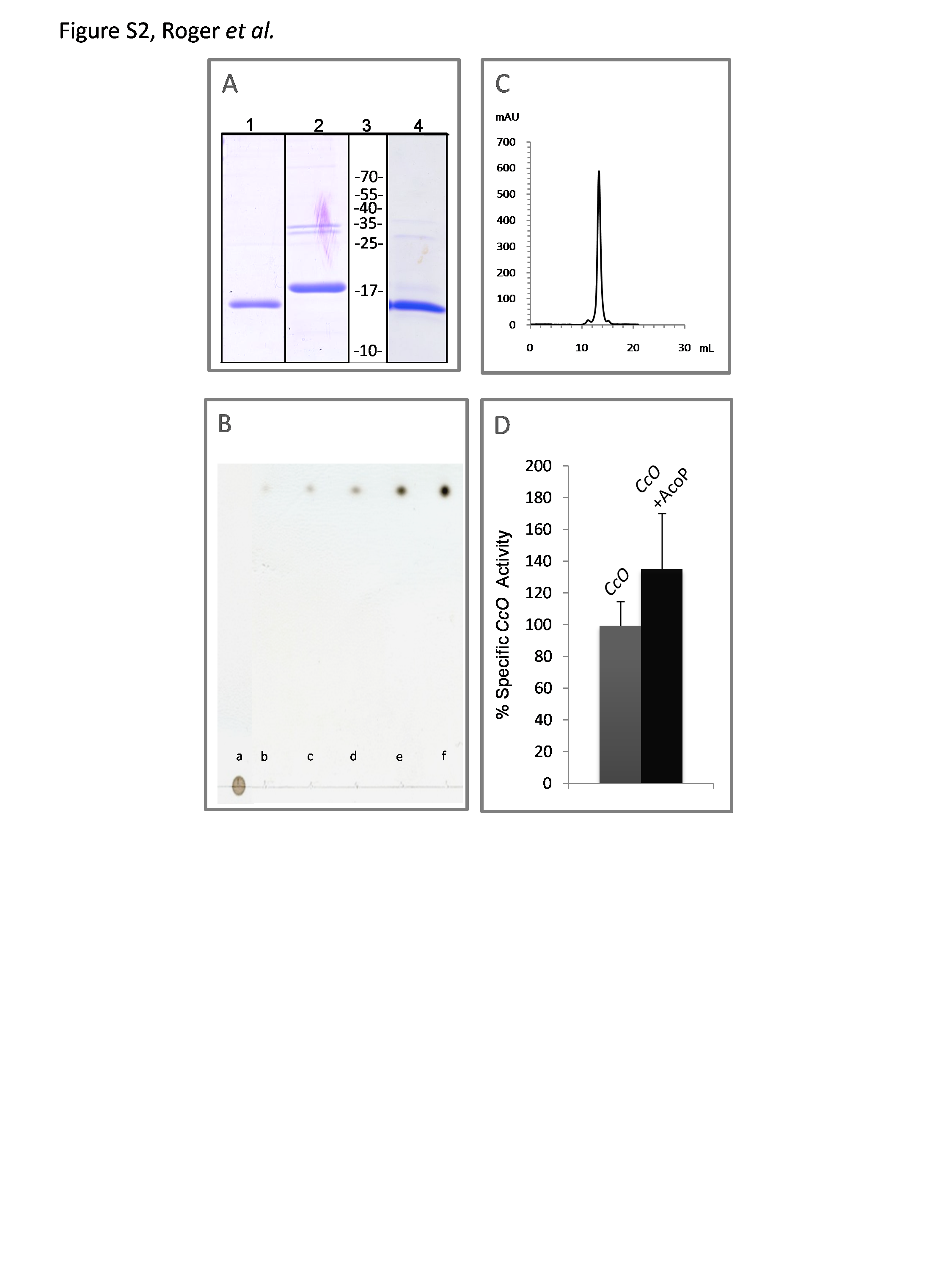

Supplement: Figure S2 — Analytical procedures. (A) Protein purity. Coomassie blue staining of purified azurin (lane 1), rusticyanin (lane 2) and AcoP from E. coli (lane 4) run on 15% SDS-PAGE. 15 µg of proteins were loaded on the gel. Molecular mass markers are indicated in lane 3. (B) Thin layer chromatography of purified AcoP. 5 µL of sample was loaded (lane a). We can estimate that the DDM amount in AcoP sample is very low (less than 0.02%), by direct comparison with increasing quantities of DDM (1, 2, 5 10 and 20 µg, lane b to f). (C) Gel filtration of purified AcoP. 3 mg of sample was loaded on S 75 gel filtration column using an ÄKTA basic FPLC setup. A single major molecular peak on the chromatogram was obtained. Fractions from this peak gave a single band on SDS-PAGE. (D) Effect of recombinant AcoP on the cytochrome c oxidase (CcO) activity from A. ferrooxidans. The relative 100% corresponds to the activity of a partially destabilized cytochrome c oxidase from which the specific activity is 0.3 µmol/mg/min. AcoP alone does not present any cytochrome c oxidase activity. The addition of recombinant, reconstituted holo-AcoP (black bar) has a positive effect on the cytochrome c oxidase activity compared to the addition of equivalent amounts of buffer (dark grey bar). Results presented correspond to an average of three experiments. (TIF) [file pone.0098941.s002.tif]

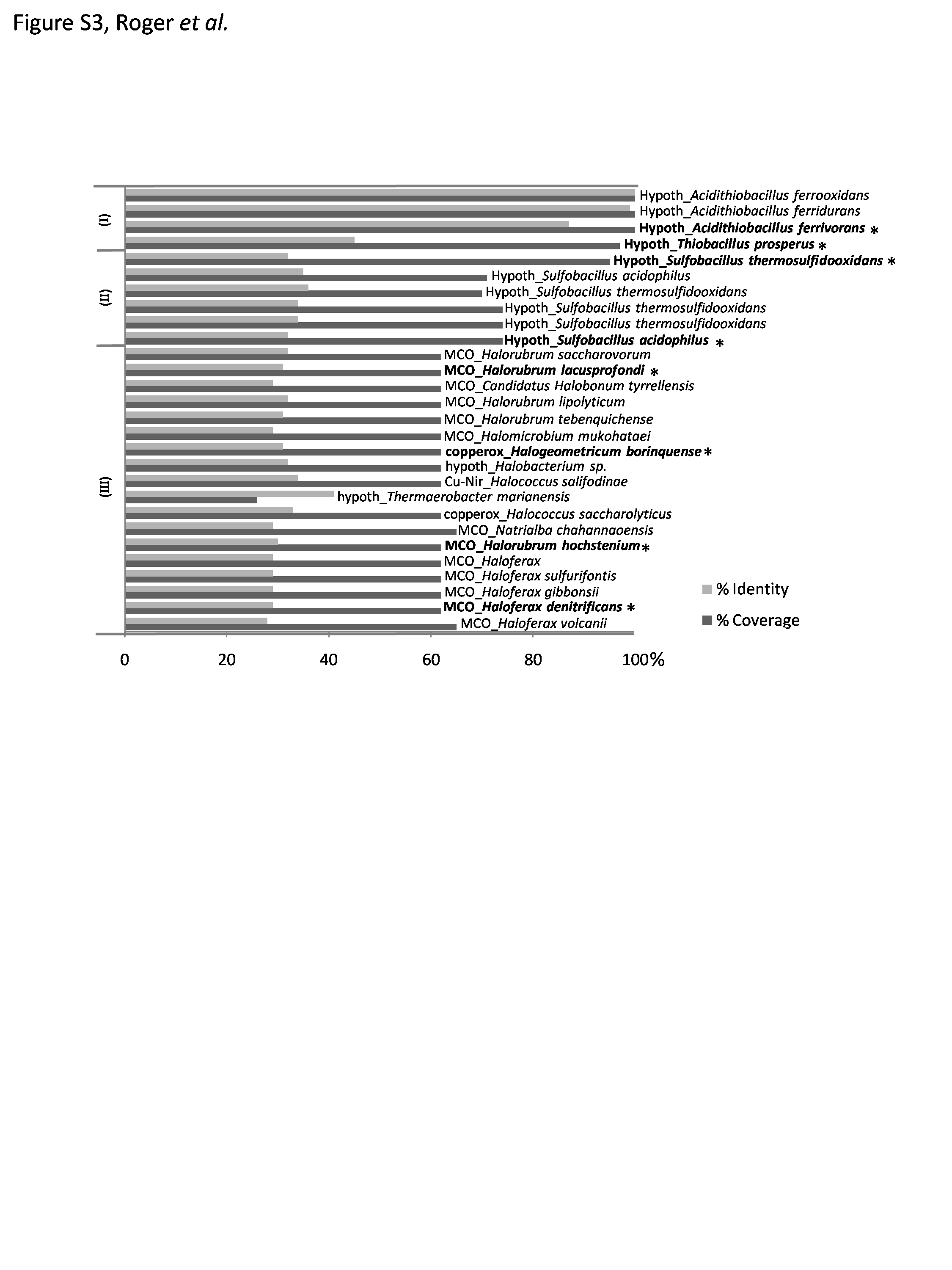

Supplement: Figure S3 — Distribution of sequence coverage and identity for 28 AcoP homologues. PSI-Blast was run on http://blast.ncbi.nlm.nih.gov using the AcoP sequence as a template (NCB Accession: YP_002427513). Dark and light grey bars represent the percentage of sequence coverage and identity respectively. Group (I) and (II) include hypothetical proteins from acidophiles with two different scores for sequence coverage and identity (e-values from 3e-132 to 1e-46 and from 8e-20 to 1e-13, respectively); (III) includes N-terminal domains of putative copper and multicopper oxidases (e-values from 1e-4 to 1e-3). (*) correspond to sequences used for multiple sequence alignment (see Figure 1). Abbreviations used: Hypoth: hypothetical protein; MCO: multicopper oxidase; copperox: copper oxidase; Cu-Nir: Nitrite reductase copper containing protein. (TIF) [file pone.0098941.s003.tif]

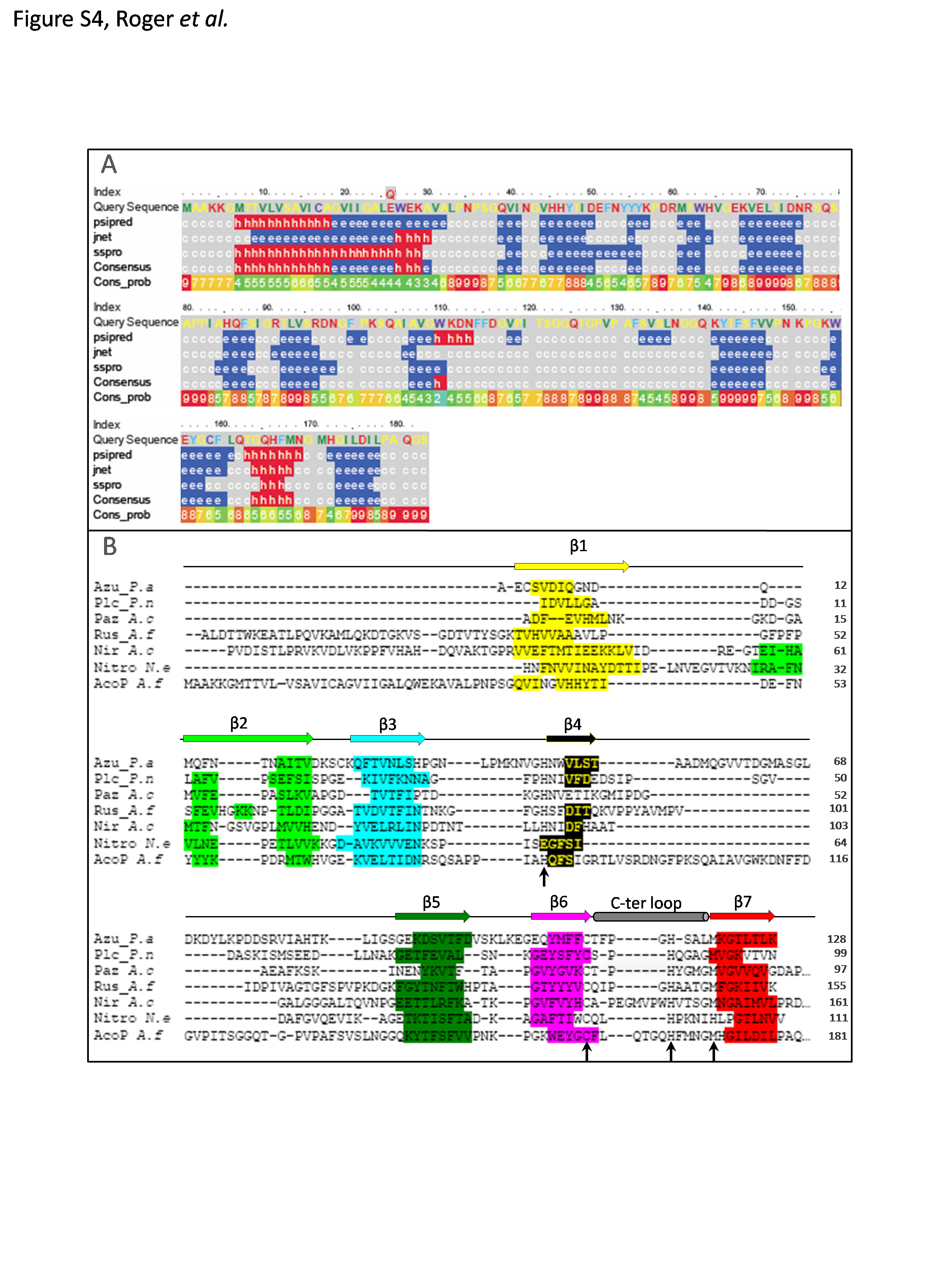

Supplement: Figure S4 — Sequence alignement based on secondary structure elements of AcoP with cupredoxins of known 3D structure. (A) Secondary structure prediction of AcoP using PHYRE [39]. Alpha helices (h), beta sheets (e) and random coiled (c) are colored in red, blue and grey, respectively. Two bottom lines: consensus sequence and consensus probability (“cons_prob”); 9 (red) high prediction probability; 2 (cyan) the lowest probability in this prediction. A metaserver approach was required for this secondary structure prediction because we obtained very different result using single software analysis. Accordingly, a very limited set of secondary structure elements has been predicted with high confidence. (B) Sequence alignement of secondary structure elements of AcoP (predicted using PHYRE, see A) with well-known cupredoxins. Secondary elements of the cupredoxin fold are reported on top. Yellow, light green, cyan, black, dark green, magenta and red arrows: β-Strand 1 to 7. The C-terminal a-helical loop (C-ter loop), between the second and fourth copper ligand, is also reported (grey cylinder). Vertical black arrows indicate copper binding ligands. Aligned sequence: azurin from Pseudomonas aeruginosa (Azu_P.a), PDB # 1E65; plastocyanin from Populus nigra (Plc_P.n), PDB # 1PLC; pseudoazurin from Achromobacter cycloclastes (Paz_A.c), PDB # 1ZIA; rusticyanin from Acidithiobacillus ferrooxidans (Rus_A.f), PDB # 2CAK are well studied single-domain blue copper proteins with known structure. Nitrite reductase from Achromobacter cycloclastes (Nir_A.c), PDB # 2BWI is a model of a green copper center and it belongs to the multi-domain cupredoxin subfamily. Nitrosocyanin from Nitrosomonas europaea (Nitro_N.e), PDB # 1IC0A belongs to the red cupredoxin subfamily. (TIF) [file pone.0098941.s004.tif]

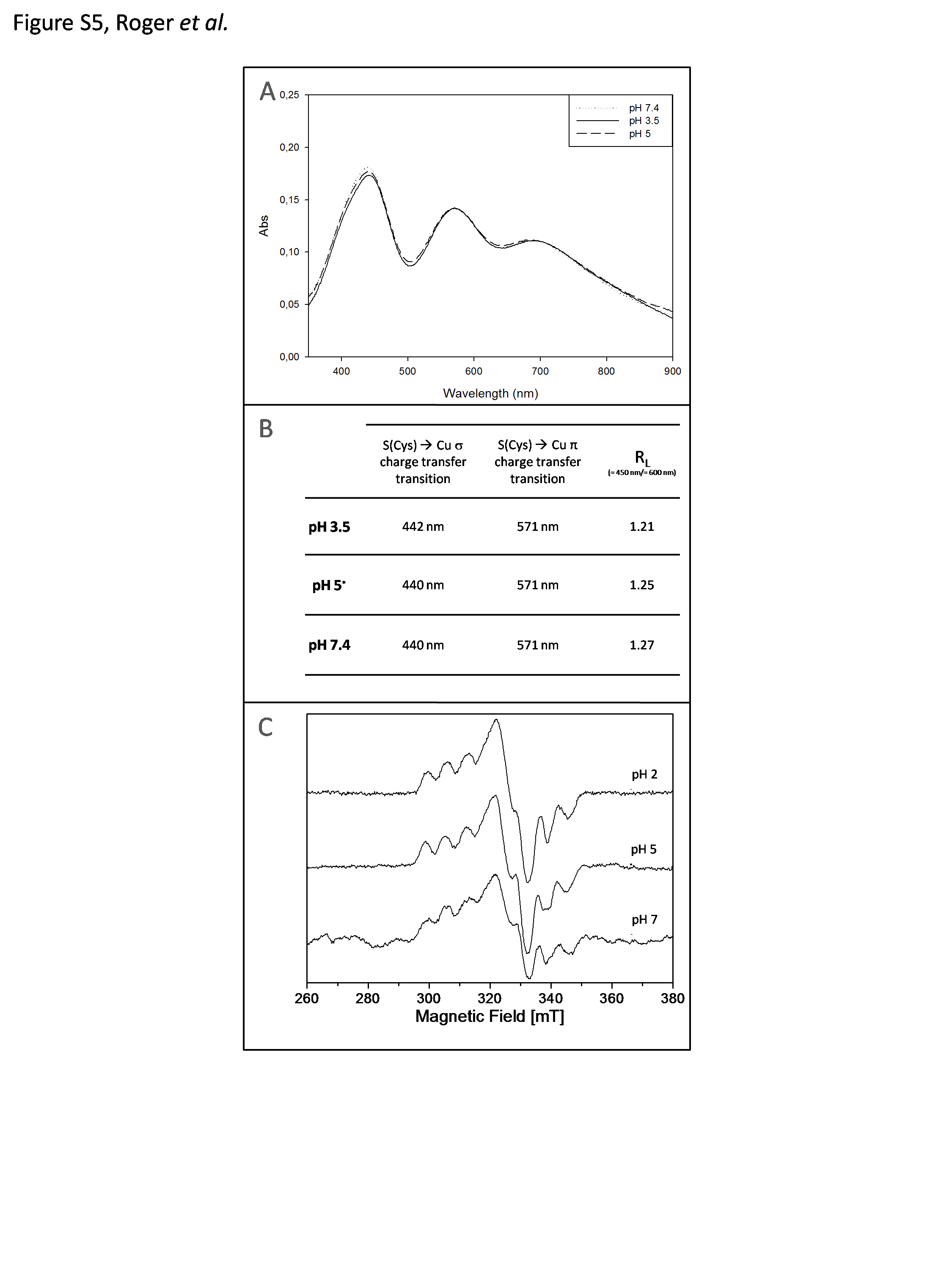

Supplement: Figure S5 — pH-independent spectroscopic behavior of AcoP. (A) UV-Visible spectra of AcoP (50 µM) in universal buffer (50 mM sodium acetate, 25 mM MOPS, 25 mM MES) at pH 3.5 (solid line); pH 5.0 (dashed-line) and pH 7.4 (dotted-line). (B) Spectroscopic parameters of AcoP at pH 3.5, pH 5.0, and pH 7.4. (*)Values obtained in the universal buffer pH 5 show a slight shift (2 nm) if compared to the ones from buffer B pH 5 (Table 1). (C) Frozen solution X-band EPR spectra of AcoP at pH 2.0, 5.0 and 7.0. Experimental conditions: T = 15 K, microwave frequency 9.48 GHz, modulation amplitude 2 mT, microwave power 1 mW. (TIF) [file pone.0098941.s005.tif]

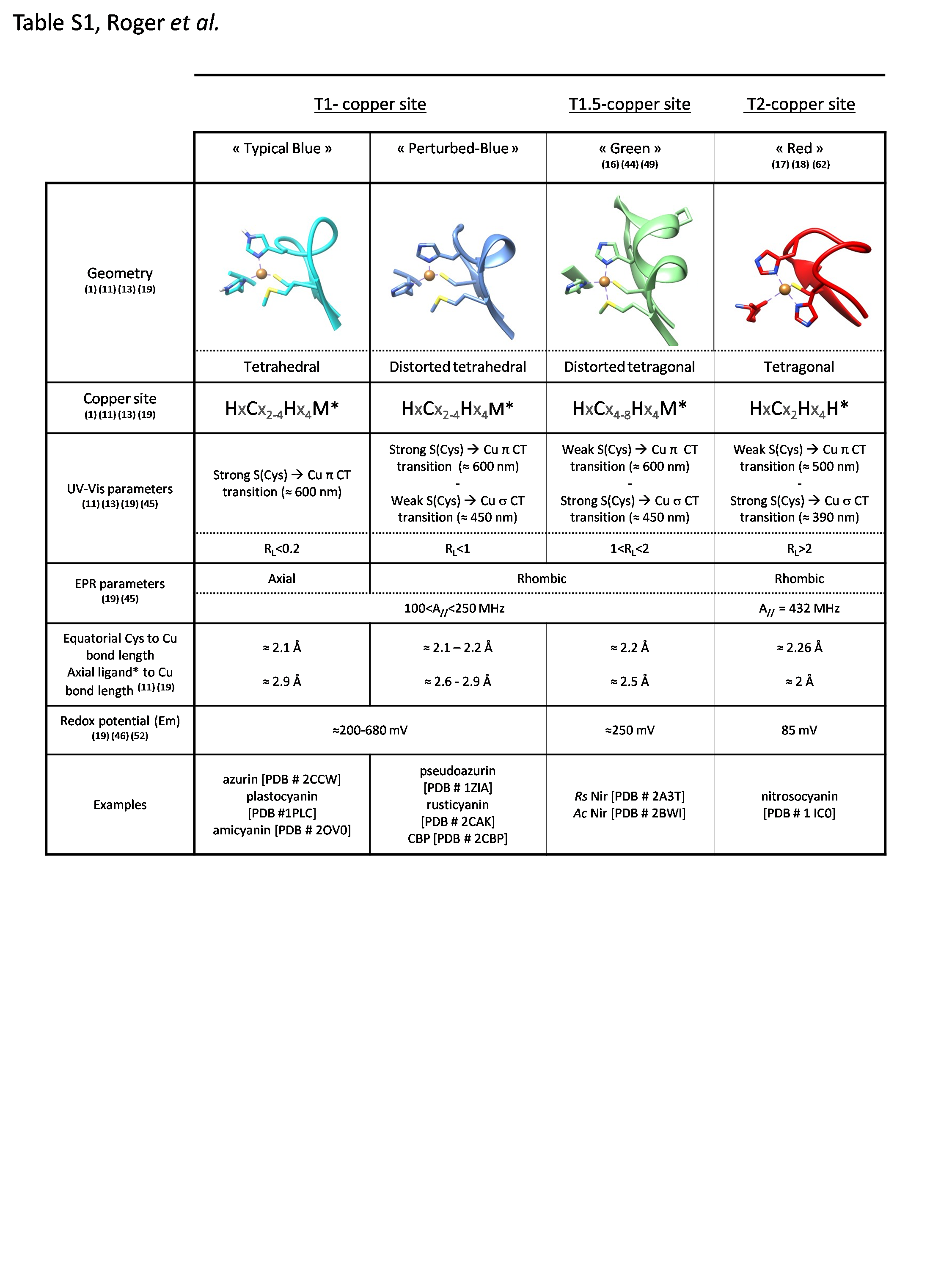

Supplement: Table S1 — Overview of structural and spectroscopic properties of some “Blue”, Green” and “Red” copper sites. (*) refers to the axial ligand. The axial residue is usually a methionine, however, some T1-copper site (such as stellacyanin) can have a glutamine residue as axial ligand [59]. (TIF) [file pone.0098941.s006.tif]
